# Retrograde apoptotic signaling by the p75 neurotrophin receptor

**DOI:** 10.1042/NS20160007

**Published:** 2017-02-24

**Authors:** Amrita Pathak, Bruce D. Carter

**Affiliations:** Department of Biochemistry and Vanderbilt Brain Institute, Vanderbilt University School of Medicine, Nashville, TN, U.S.A.

**Keywords:** apoptosis, axonal transport, JNK, nerve growth factor, neurotrophin, NRIF

## Abstract

Neurotrophins are target-derived factors necessary for mammalian nervous system development and maintenance. They are typically produced by neuronal target tissues and interact with their receptors at axonal endings. Therefore, locally generated neurotrophin signals must be conveyed from the axon back to the cell soma. Retrograde survival signaling by neurotrophin binding to Trk receptors has been extensively studied. However, neurotrophins also bind to the p75 receptor, which can induce apoptosis in a variety of contexts. Selective activation of p75 at distal axon ends has been shown to generate a retrograde apoptotic signal, although the mechanisms involved are poorly understood. The present review summarizes the available evidence for retrograde proapoptotic signaling in general and the role of the p75 receptor in particular, with discussion of unanswered questions in the field. In-depth knowledge of the mechanisms of retrograde apoptotic signaling is essential for understanding the etiology of neurodegeneration in many diseases and injuries.

## Introduction

Neuronal apoptosis is a normal part of development, required for the establishment of the proper circuitry of the nervous system. However, the abnormal death of neurons is associated with a variety of neurodevelopmental disorders and neurodegenerative conditions. The regulation of neuronal survival is controlled, in part, by the neurotrophin family of trophic factors, which includes nerve growth factor (NGF), brain-derived neurotrophic factor (BDNF), and neurotrophin (NT)-3 and NT4, as well as their proforms. The mature forms of NTs bind selectively to members of the Trk family of tyrosine kinase receptors (TrkA, B, and C); they promote survival and differentiation, and modulate synaptic function [1]. In contrast, all the mature and pro-neurotrophins bind to the p75 NT receptor, which is a pleiotropic signaling molecule that can promote survival and yet can also induce apoptosis, among other functions [2,3]. The receptor p75 can interact with a variety of co-receptors, and the nature of the signal generated by ligand binding depends on the specific receptor complex, e.g. survival signals are generated when a mature NT binds to a complex of its cognate Trk receptor and p75 [2,4]. In contrast, NT binding to p75 alone or pro-neurotrophin binding to a complex of p75 and members of the Vps10p family of sorting receptors, typically activates proapoptotic or axon degeneration signals [5].

## Mechanism of p75-mediated apoptosis

The p75 receptor belongs to the tumor necrosis factor (TNF) receptor (TNFR) superfamily of death receptors. Similar to other members of the TNFR superfamily, p75 is a type I, single-transmembrane, domain protein with a cysteine-rich extracellular domain (ECD) and a death domain (DD) in its intracellular tail. The structure of the p75 DD is similar to that found in several other members of the TNFR superfamily [6]; however, p75 exists in a dimeric conformation, linked by a disulfide bond in its transmembrane region [7], whereas most members of this superfamily form trimers. The DD is linked to the transmembrane domain by a flexible juxtamembrane region, and studies using cultured primary neurons have implicated both the DD [8] and the juxtamembrane domain [9] in p75-mediated cell death.

On ligand binding, p75 signals by recruiting various adaptor proteins to its intracellular domain (ICD), leading to activation of several downstream pathways, depending on the specific ligand and cellular context. The p75 signaling pathways have been thoroughly reviewed elsewhere [3] and are just summarized here. Adaptors, such as TNFR-associated factor 6 (TRAF6) [10] and Rip2 [11], associate with p75 and induce activation of the transcription factor nuclear factor κ-light-chain-enhancer of activated B-cells (NF-κB), which has been linked to the promotion of survival [3,12,13]. Binding of other adaptors, including Rho GDP-dissociation inhibitor (RhoGDI) [14] and Trio [15], result in activation of RhoA and reduced Rac activity, which promotes growth cone collapse and neurite retraction. Finally, several receptor-associated factors, such as TRAF6 (in the absence of Rip2) [10,11], NTR-interacting factor (NRIF) [16], and NRAGE [17], bind to p75’s ICD and promote activation of c-Jun N-terminal kinase (JNK). JNK activation results in phosphorylation of a variety of substrates, including the transcription factor c-Jun and members of the Bcl-2 family [18] that induce apoptosis. However, unlike death induced by growth factor withdrawal, c-Jun itself is dispensable for p75-mediated apoptosis, suggesting that the other substrates have a more critical role [19].

**Figure 1:**
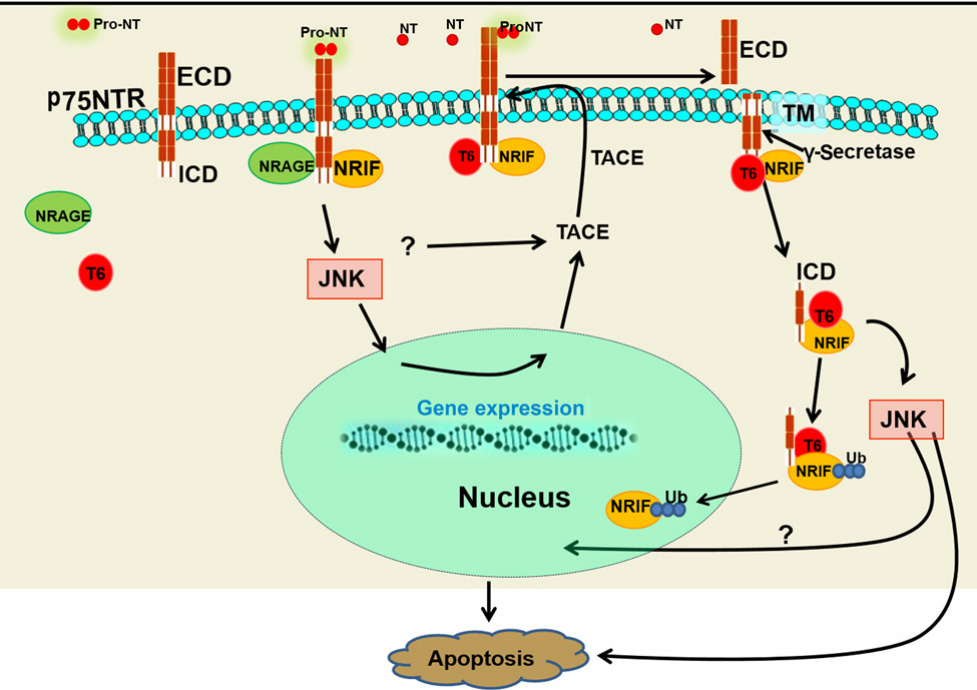
Apoptotic signaling mechanisms activated by the p75 receptor Proapoptotic ligands bind to preexisting p75 dimers, resulting in recruitment of adaptor proteins such as NRAGE, TRAF6, and NRIF, leading to stimulation of JNK. The activation of JNK induces p75 proteolysis, first in the ECD by TACE, and then in the transmembrane domain by the γ-secretase complex. JNK can transcriptionally up-regulate TACE; however, there also appear to be other, more rapid mechanisms for stimulating this protease. The cleavage by γ-secretase releases p75’s ICD, resulting in a complex of the ICD, NRIF, a DNA-binding protein, and TRAF6 (T6), which promotes NRIF ubiquitylation (Ub). Ubiquitylated NRIF translocates to the nucleus and mediates apoptosis. In addition, this complex also leads to apoptosis through prolonged JNK activity.

Stimulation of JNK was also shown to up-regulate the metalloprotease TNFα-converting enzyme (TACE/ADAM17) [20], which cleaves the ECD of the receptor. In sympathetic neurons, JNK activation transcriptionally up-regulated TACE, which took several hours [20]. However, in other contexts, not associated with apoptosis, TACE-mediated cleavage of p75 in response to various stimuli is much faster, suggesting other mechanisms for activation of this protease [21–24]. Proteolysis of p75 by TACE is followed by γ-secretase-mediated cleavage within the transmembrane domain of the receptor, releasing the ICD [23,24]. Liberation of the ICD facilitates translocation of NRIF, a DNA-binding protein that associates with the p75 ICD, into the nucleus, which is required for receptor-mediated apoptosis in several cell types [3,25,26] (Figure 1).

## Retrograde apoptotic signaling by the p75 receptor

Although some of the signaling mechanisms activated by p75 are beginning to be revealed, their spatial localization remains largely unexplored and may play an important role in determining the ultimate cellular response. Neurons are highly polarized cells with axons that often extend great distances from the soma. Therefore, some signals may be locally confined to regulate process retraction or growth, whereas other signals may be conveyed back to the cell soma and affect survival. NTs are classically defined as target-derived factors and interact with axons innervating or synapsing on to their targets. Therefore, to affect transcription within the cell nucleus and/or survival, NT signals must be retrogradely transported back to the cell body.

Long-range signaling in axons occurs via transport along microtubules, driven by the motor protein dynein for retrograde movement and kinesin for anterograde movement. These molecular motors are large protein complexes that assemble on vesicles, and both motor types can be found on a single vesicle [27]. The ultimate direction in which the vesicle moves is thought to depend on which motor complex is more abundant and/or how it is regulated, but is still an active area of investigation.

As p75-mediated apoptosis requires signaling to the nucleus, a retrograde transport system must exist within the neuron to bring the signal from the activated receptor at the cell membrane to the nucleus. Therefore, it is likely that there is a similar mechanism for transporting p75 signals from distal axons back to the cell body. One of the first indications that selective activation of p75 in distal axons could induce retrograde apoptotic signaling came from studies of neurons in the isthmo-optic nucleus (ION) of the chick. The ION neurons innervate the retina, and von Bartheld et al. [28] demonstrated that NGF could be transported by p75 from the eye back to the ION, resulting in neuronal cell death. Since then, there have been few studies examining the role of p75 retrograde apoptotic signaling during normal development. Taylor et al. [29] provided evidence of p75-mediated apoptosis during development of motor neurons in the chick embryo. They demonstrated that muscle cells release pro-BDNF, which induced apoptosis of spinal motor neurons in culture, and the developmental apoptosis of these neurons *in vivo* was prevented by antibodies blocking p75; Sortilin (a Vps10p co-receptor) or pro-BDNF. These results suggest that an apoptotic signal is transported, by p75 and Sortilin, from the muscles back to the motor neuron soma. Although such p75-mediated retrograde apoptotic signaling has not been reported for motor neurons in mammals, it is notable that there is reduced developmental apoptosis of spinal neurons in the early stages of development of *p75^−/−^* mice [30]. In addition, treatment of cultured rodent motor neurons with NGF, which binds only p75 and none of the Trks in these neurons, induced cell death [31,32].

Retrograde apoptotic signaling by p75 has been suggested in several injury models. After axotomy of the corticospinal tract, respective motor neurons with their cell bodies present in the cortex undergo p75-dependent apoptosis [33]. Similarly, p75 has been implicated in the death of sensory neurons in the dorsal root ganglia [34], spiral ganglion neurons in the inner ear [35], and olfactory receptor neurons [36] after injury to distal axons. Interestingly, p75 is up-regulated in a wide variety of neuronal injuries and neuropathologies [37]. Although direct evidence linking the receptor to the associated neurodegeneration is currently limited to just a few conditions, p75 may contribute to many of these pathologies, possibly through retrograde degenerative signaling.

Using an *in vitro* culture system, Teng and colleagues provided convincing evidence for neuronal death through a p75-mediated retrograde signal [38]. They cultured sympathetic neurons in compartmentalized cultures, which create a physical barrier between the cell soma and the distal axons. Addition of pro-NT3 exclusively to the distal axons induced neuronal death, which was detected in the cell bodies. Importantly, neurons from *p75^−/−^* mice were resistant to pro-NT3, thereby demonstrating that the receptor was required for the resulting apoptosis. These investigators pointed out that many of the targets innervated by sympathetic neurons produce NT3, so they must also produce pro-NT3, because it is a precursor. It is not yet clear whether pro-NT3 is released from the target organs at sufficient levels to induce neuronal apoptosis, but it was suggested that this pro-NT could function as a developmental apoptotic signal that is retrogradely transported.

## NT receptor internalization

Intra-axonal trafficking of receptors and their signaling components typically occurs via vesicular transport. Retrograde NT signaling has been extensively characterized for pro-survival signaling through the Trk receptors. Considerable evidence indicates that, on NT binding to a Trk receptor in the axon, the ligand-bound receptor is internalized and retrogradely transported in what has been termed a ‘signaling endosome’ (for reviews see [39–44]). This term was coined after the finding that a remarkable number of Trk signaling components remain associated with the endosome as it is transported back to the cell body [45]. Multiple studies from various groups using different neuronal systems have supported the signaling endosome model; however, even 15 years after its inception, a general consensus on the functional definition of a signaling endosome, the key signaling components present in it, and the nature of the vesicle/endosome being trafficked is lacking [46,47]. In addition, other mechanisms have also been proposed by which Trk receptors signal retrogradely, e.g. transport of the Trk signal, independent of NT internalization [48] or binding of a ligand [49], and propagating waves of Trk phosphorylation along the plasma membrane [50]. Thus, despite a general understanding of retrograde NT signaling by Trk receptors, there are still many unanswered questions.

Although the molecular components of retrograde signaling by the p75 receptor remain to be investigated, some of the mechanisms of its internalization are starting to be elucidated. Similar to many aspects of p75 signaling, the pathways of internalization depend on the cellular context. In general, p75 is endocytosed with much slower kinetics after ligand binding than the Trk receptors [51,52], although the exact rate depends on the type of neuron, e.g. p75 internalization is faster in hippocampal than in sympathetic neurons [53]. Endocytosis of p75 has been reported to occur via both clathrin-dependent and -independent mechanisms in sympathetic neurons [54,55] and motor neurons [56], although the functional significance of using these two routes is not known. After ligand-induced internalization in PC12 cells and sympathetic neurons, it was reported that p75 avoided the lysosome and was trafficked to Rab11^+^ recycling endosomes or multivesicular bodies (MVBs), where some of the receptor was exocytosed [55]. It is interesting that, in the chick ION, retrograde p75-mediated cell death correlated with accumulation of NGF in the neuronal soma in MVBs [57], suggesting that retrograde transport of p75 occurs in MVBs. In contrast, in motor neurons p75 is first trafficked into Rab5^+^ early endosomes, then into Rab7^+^ endosomes for retrograde transport [56,58]. The type of vesicle responsible for trafficking p75 from the axon to the soma may depend on the ligand and the type of neuron, which could also affect which signals end up at the cell body.

It is important to note that the studies examining p75 trafficking have been carried out using labeled ligands or antibodies to the ECD. As the receptor undergoes proteolytic processing, it is unclear whether the ECD and the ICD traffic together or are separated. The Bronfman group demonstrated that, in PC12 cells, p75 is cleaved by TACE at the cell surface, releasing the ECD, and then internalized; γ-secretase cleavage occurs on endosomes [22]. However, this processing of p75 was actually triggered by Trk activation in this cell line and was not associated with any apoptosis. Therefore, whether the cleavages would occur in the same location in neurons after ligand binding to p75 remains an open question. Moreover, if the ECD is released from the axonal surface, then the previous studies of p75 trafficking could be measuring only movement of the full-length receptor, which may send different signals back to the soma compared with the released ICD.

## Retrograde degenerative signaling

The mechanisms by which the axonally activated p75 receptor communicates with the neuron cell body have yet to be determined. However, a number of degenerative retrograde signals after injury or trophic factor deprivation have been identified, and there may be some overlap with p75-dependent processes, so it is worth some discussion here.

After axotomy, one of the first responses at the injury site is an increase in intracellular calcium, which can propagate along the axon, back to the soma [59,60]. Although elevated calcium is important for repair at the damage site [61], if the levels become too high in the soma, they can lead to cell death through a mechanism analogous to excitotoxicity. Locally increased calcium also activates the dual leucine zipper kinase (DLK), a mitogen-activated protein 3-kinase (MAP3K) that can activate the MAP kinases, JNK and p38 [62]. DLK forms a complex with the scaffold protein, JNK-interacting protein 3 (JIP3), which leads to activation of JNK [63]. These authors also demonstrated that sensory neurons from *dlk^−/−^* mice fail to activate JNK after trophic factor withdrawal, and exhibit a dramatic decrease in the level of apoptosis and axon degeneration. Furthermore, naturally occurring cell death in the developing dorsal root ganglia and spinal motor neurons was significantly reduced in the *dlk^−/−^* mice [63,64]. Ghosh et al. [63] suggested that a DLK–JNK–JIP3 complex forms in axons deprived of trophic factor and is retrogradely transported to the neuron cell body, where it promotes cell death.

JNK and JIP3 were shown to be constitutively associated in peripheral axons and underwent both retrograde and anterograde transport [65]. However, after nerve injury, JIP3 preferentially associated with dynein through binding to dynactin, a component of the dynein complex, resulting in predominantly retrograde transport of JNK–JIP3. Similar to retrograde signaling after trophic factor withdrawal, the retrograde transport of JIP3 after nerve injury was also dependent on DLK [66], suggesting that a DLK–JNK–JIP3 complex also transmits an injury signal back to the cell soma. Paradoxically, however, this complex does not induce neuronal degeneration, but is necessary for nerve regeneration [66]. How this retrograde signaling complex can mediate such opposing effects is unclear; however, nerve injury also induces the DLK-dependent retrograde transport of the transcription factor STAT3 [66]. Therefore, specific components of the retrograde complex, such as STAT3, may function as a critical switch in determining the neuronal response.

Other proapoptotic signals have also been reported to be retrogradely transported, e.g. apoptosis of sympathetic neurons induced by withdrawal of NGF from distal axons required glycogen synthase kinase 3β (GSK3) activity in the axons, leading the authors to suggest that GSK3 functioned as a retrograde signal [67]. However, they did not directly investigate the components of the retrograde signaling complex or track GSK3 transport. The proapoptotic protease caspase 8 has also been implicated in retrograde degenerative signaling after removal of the olfactory bulb, which damages the axons of olfactory receptor neurons and leads to neuronal death [36]. The active form of caspase 8 was associated with dynactin, and disruption of microtubules in the axons prevented the accumulation of active caspase 8 at the neuronal somata and the associated apoptosis, indicating that the protease was retrogradely transported. It is interesting that deletion of p75 also rescued the olfactory neurons, suggesting that the receptor is a key contributor to the apoptotic mechanism. How p75 functions in this degenerative process is not, however, clear because evidence suggests that p75 does not activate caspase 8 [68].

In addition to mediating cell death, recent findings have highlighted a role for proapoptotic caspases in axonal degeneration (reviewed by Geden and Deshmukh [69]). Several studies have revealed that trophic factor withdrawal triggers local activation of caspases 3, 6, and 9 in axons, resulting in their breakdown [70–72]. This degenerative process can occur independent of apoptosis, as long as the neuronal soma is receiving trophic support from another source (e.g. at the cell body). Such selective pruning allows circuit refinement without neuronal loss. Surprisingly, a recent study demonstrated that even localized axon degeneration requires retrograde signaling to the soma [73]. On receipt of a degenerative retrograde signal, there is a transcriptional up-regulation of proapoptotic factors, such as the p53-up-regulated modulator of apoptosis (PUMA), resulting in anterograde transport of signals to promote the breakdown of axons. The nature of the retrograde and anterograde degenerative signal in this context has yet to be defined, but may involve p75. Selective activation of p75 in distal axons can induce localized degeneration without causing apoptosis [74] and is an essential process for normal axon pruning [75]. Whether p75 or its signaling partners are components of the retrograde degenerative signal processed back at the soma remains to be determined.

The receptor p75 has also been implicated in retrograde degenerative signaling in motor neurons of superoxide dismutase 1 mutant mice (SOD1^G93A^), which undergo axon degeneration and eventual motor neuron apoptosis and have been used as a model for amyotrophic lateral sclerosis (ALS) [76]. Initially, p75 was suggested to be involved in the neurodegeneration in ALS patients and SOD1^G93A^ mice based on the up-regulation of the receptor in the affected motor neurons [77,78]. However, crossing the SOD1^G93A^ mice with p75*^−/−^* mice provided only partial protection in females [79]. These results are complicated by the multifaceted role of the receptor in other cells that could affect motor neuron viability (e.g. Schwann cells [80] and astrocytes [81,82]). Nevertheless, acute inhibition [83–85] or knockdown of p75 [86] in the SOD1^G93A^ mice was shown to reduce the neurodegeneration. Furthermore, the p75 ICD was found to associate with the retrograde motor dynein in the nerves from these mice, suggesting that it is retrogradely transported in the neurons [76]. The authors pulled down dynein from motor axons in wild-type and SOD1^G93A^ mice, and then surveyed the associated proteins using mass spectrometry. In wild-type, healthy axons, dynein complexes included many pro-survival signals, such as activated Trk and ERKs; however, in the SOD1^G93A^ axons, there were many proapoptotic factors such as caspase 8, activated JNK, and the ICD fragment of p75. Importantly, inhibition of the retrograde transport of the proapoptotic factors could rescue the SOD1^G93A^-expressing motor neurons. Based on their results, the authors proposed that the retrograde transport of these proapoptotic factors could contribute to the neurodegeneration associated with ALS.

## Conclusions

**Figure 2:**
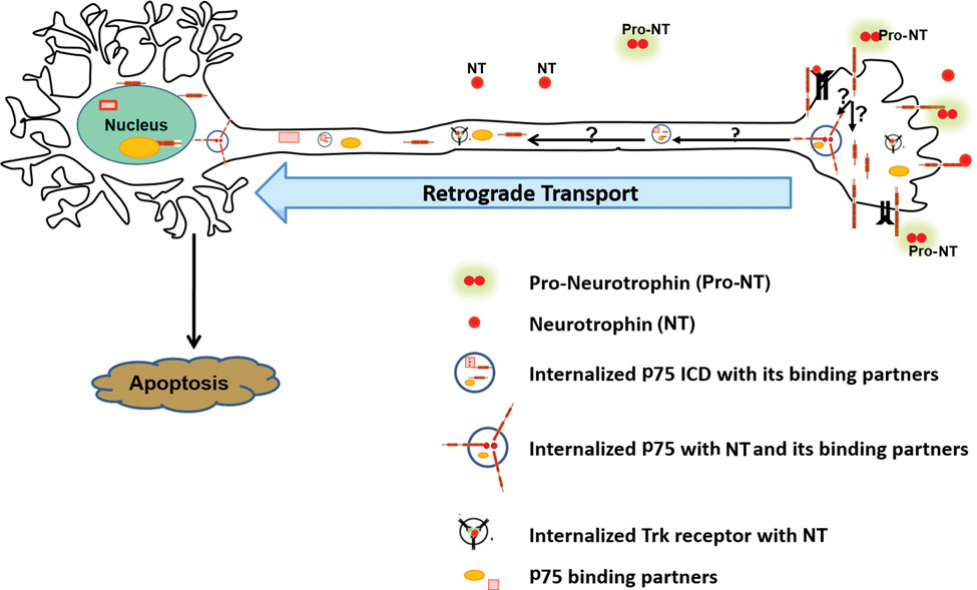
Retrograde apoptotic signaling by p75 Many questions remain to be answered about retrograde signaling by p75 (indicated by question marks), including the mechanisms of internalization, the identity of the transport vesicle, where the cleavage occurs, and which fragments of p75 are transported, as well as which components are present in the retrograde signaling complex (e.g. various p75-binding partners).

Given the extensive length of many axons, it is not surprising that transport plays a key role in regulating neuronal survival. Many critical factors are shuttled back and forth along the axon, including trophic factors and associated signals to mitochondria and lysosomes. It is well established that disruptions in retrograde transport are associated with a number of neurodegenerative conditions, such as Alzheimer's and Huntington's diseases and a variety of motor neuron diseases, including ALS [87,88]. However, there is growing evidence that proapoptotic factors can also be retrogradely transported under various pathological conditions. The p75 receptor has been implicated in retrograde degenerative signaling in a few conditions, as discussed above; however, there remain many unanswered questions about the role of the receptor, the mechanisms of its transport, and the components of the signaling complex with which it associates (Figure 2). Understanding the mechanisms regulating the balance between pro-survival and proapoptotic retrograde signaling is essential to develop therapeutic strategies for neurodegenerative diseases.

## Abbreviations

ALS: amyotrophic lateral sclerosis
BDNF: brain-derived neurotrophic factor
DD: death domain
DLK: dual leucine zipper kinase
ECD: extracellular domain
ERK: extracellular signal-related kinase
GSK3: glycogen synthase kinase 3β
ICD: intracellular domain
ION: isthmo-optic nucleus
JIP3: JNK-interacting protein 3
JNK: c-Jun N-terminal kinase
MAP: mitogen-activated protein
MVB: multivesicular body
NGF: nerve growth factor
NRIF: NTR-interacting factor
NT: neurotrophin
NTR: NT receptor
TACE: TNFα-converting enzyme
SOD: superoxide dismutase
TNF: tumor necrosis factor
TRAF: TNFR-associated factor

## References

[1] Deinhardt K., Chao M.V. (Lewin G.R., Carter B.D., eds), (2014) Trk receptors. Neurotrophic Factors Berlin Heidelberg Springer, 103–119 10.1007/978-3-642-45106-5

[2] Chao M.V. (2003) Neurotrophins and their receptors: a convergence point for many signalling pathways. Nat. Rev. Neurosci. 4, 299–309 10.1038/nrn1078 12671646

[3] Kraemer B.R., Yoon S.O., Carter B.D. (2014) The biological functions and signaling mechanisms of the p75 neurotrophin receptor. Handb. Exp. Pharmacol. 220, 121–164 10.1007/978-3-642-45106-5 24668472

[4] Hempstead B.L., Martin-Zanca D., Kaplan D.R., Parada L.F., Chao M.V. (1991) High-affinity NGF binding requires coexpression of the trk proto-oncogene and the low-affinity NGF receptor. Nature 350, 678–683 10.1038/350678a0 1850821

[5] Meeker R., Williams K. (2015) The p75 neurotrophin receptor: at the crossroad of neural repair and death. Neural. Regen. Res. 10, 721 10.4103/1673-5374.156967 26109945 PMC4468762

[6] Liepinsh E., Ilag L.L., Otting G., Ibáñez C.F. (1997) NMR structure of the death domain of the p75 neurotrophin receptor. EMBO J. 16, 4999–5005 10.1093/emboj/16.16.4999 9305641 PMC1170134

[7] Vilar M., Charalampopoulos I., Kenchappa R.S., Simi A., Karaca E., Reversi A. et al. (2009) Activation of the p75 neurotrophin receptor through conformational rearrangement of disulphide-linked receptor dimers. Neuron 62, 72–83 10.1016/j.neuron.2009.02.020 19376068 PMC2810632

[8] Charalampopoulos I., Vicario A., Pediaditakis I., Gravanis A., Simi A., Ibáñez C.F. (2012) Genetic dissection of neurotrophin signaling through the p75 neurotrophin receptor. Cell Rep. 2, 1563–1570 10.1016/j.celrep.2012.11.009 23260665

[9] Coulson E.J., Reid K., Baca M., Shipham K.A., Hulett S.M., Kilpatrick T.J. et al. (2000) Chopper, a new death domain of the p75 neurotrophin receptor that mediates rapid neuronal cell death. J. Biol. Chem. 275, 30537–30545 10.1074/jbc.M005214200 10882742

[10] Yeiser E.C., Rutkoski N.J., Naito A., Inoue J., Carter B.D. (2004) Neurotrophin signaling through the p75 receptor is deficient in traf6-/- mice. J. Neurosci. 24, 10521–10529 10.1523/JNEUROSCI.1390-04.2004 15548667 PMC6730299

[11] Khursigara G., Bertin J., Yano H., Moffett H., DiStefano P.S., Chao M.V. (2001) A prosurvival function for the p75 receptor death domain mediated via the caspase recruitment domain receptor-interacting protein 2. J. Neurosci. 21, 5854–5863 11487608 10.1523/JNEUROSCI.21-16-05854.2001 PMC6763175

[12] Gentry J.J., Casaccia-Bonnefil P., Carter B.D. (2000) Nerve growth factor activation of nuclear factor κB through Its p75 receptor is an anti-apoptotic signal in RN22 schwannoma Cells. J. Biol. Chem. 275, 7558–7565 10.1074/jbc.275.11.7558 10713062

[13] Gentry J.J., Barker P.A., Carter B.D. (2004) The p75 neurotrophin receptor: multiple interactors and numerous functions. Prog. Brain Res. 146, 25–39 10.1016/S0079-6123(03)46002-0 14699954

[14] Yamashita T., Tohyama M. (2003) The p75 receptor acts as a displacement factor that releases Rho from Rho-GDI. Nat. Neurosci. 6, 461–467 12692556 10.1038/nn1045

[15] Deinhardt K., Kim T., Spellman D.S., Mains R.E., Eipper B.A., Neubert T.A. et al. (2011) Neuronal growth cone retraction relies upon proneurotrophin receptor signaling through Rac. Sci. Signal. 4, ra82 10.1126/scisignal.2002060 22155786 PMC3360552

[16] Linggi M.S., Burke T.L., Williams B.B., Harrington A., Kraemer R., Hempstead B.L. et al. (2005) Neurotrophin receptor interacting factor (nrif) is an essential mediator of apoptotic signaling by the p75 neurotrophin receptor. J. Biol. Chem. 280, 13801–13808 10.1074/jbc.M410435200 15668238

[17] Bertrand M.J.M., Kenchappa R.S., Andrieu D., Leclercq-Smekens M., Nguyen H.N.T., Carter B.D. et al. (2008) NRAGE, a p75NTR adaptor protein, is required for developmental apoptosis in vivo. Cell Death Differ. 15, 1921–1929 10.1038/cdd.2008.127 18772898 PMC2735073

[18] Costa C.R.D., Villadiego J., Sancho R., Fontana X., Packham G., Nateri A.S. et al. (2010) Bag1-L is a phosphorylation-dependent coactivator of c-Jun during neuronal apoptosis. Mol. Cell Biol. 30, 3842–3852 10.1128/MCB.01610-09 20516211 PMC2916400

[19] Palmada M., Kanwal S., Rutkoski N.J., Gustafson-Brown C., Johnson R.S., Wisdom R. et al. (2002) c-jun is essential for sympathetic neuronal death induced by NGF withdrawal but not by p75 activation. J. Cell Biol. 158, 453–461 10.1083/jcb.200112129 12163468 PMC2173823

[20] Kenchappa R.S., Tep C., Korade Z., Urra S., Bronfman F.C., Yoon S.O. et al. (2010) p75 Neurotrophin receptor-mediated apoptosis in sympathetic neurons involves a biphasic activation of JNK and up-regulation of tumor necrosis factor-α-converting enzyme/ADAM17. J. Biol. Chem. 285, 20358–20368 10.1074/jbc.M109.082834 20421303 PMC2888447

[21] Domeniconi M., Zampieri N., Spencer T., Hilaire M., Mellado W., Chao M.V. et al. (2005) MAG induces regulated intramembrane proteolysis of the p75 neurotrophin receptor to inhibit neurite outgrowth. Neuron 46, 849–855 10.1016/j.neuron.2005.05.029 15953414

[22] Urra S., Escudero C.A., Ramos P., Lisbona F., Allende E., Covarrubias P. et al. (2007) TrkA receptor activation by nerve growth factor induces shedding of the p75 neurotrophin receptor followed by endosomal γ-secretase-mediated release of the p75 intracellular domain. J. Biol. Chem. 282, 7606–7615 10.1074/jbc.M610458200 17215246

[23] Kanning K.C., Hudson M., Amieux P.S., Wiley J.C., Bothwell M., Schecterson L.C. (2003) Proteolytic processing of the p75 neurotrophin receptor and two homologs generates C-terminal fragments with signaling capability. J. Neurosci. 23, 5425–5436 12843241 10.1523/JNEUROSCI.23-13-05425.2003 PMC6741218

[24] Jung K.-M., Tan S., Landman N., Petrova K., Murray S., Lewis R. et al. (2003) Regulated intramembrane proteolysis of the p75 neurotrophin receptor modulates its association with the TrkA receptor. J. Biol. Chem. 278, 42161–42169 10.1074/jbc.M306028200 12913006

[25] Kenchappa R.S., Zampieri N., Chao M.V., Barker P.A., Teng H.K., Hempstead B.L. et al. (2006) ligand-dependent cleavage of the p75 neurotrophin receptor is necessary for NRIF Nuclear translocation and apoptosis in sympathetic neurons. Neuron 50, 219–232 10.1016/j.neuron.2006.03.011 16630834

[26] Volosin M., Trotter C., Cragnolini A., Kenchappa R.S., Light M., Hempstead B.L. et al. (2008) Induction of proneurotrophins and activation of p75NTR-mediated apoptosis via neurotrophin receptor-interacting factor in hippocampal neurons after seizures. J. Neurosci. 28, 9870–9879 10.1523/JNEUROSCI.2841-08.2008 18815271 PMC2578816

[27] Maday S., Twelvetrees A.E., Moughamian A.J., Holzbaur E.L.F. (2014) Axonal transport: cargo-specific mechanisms of motility and regulation. Neuron 84, 292–309 10.1016/j.neuron.2014.10.019 25374356 PMC4269290

[28] von Bartheld C.S., Kinoshita Y., Prevette D., Yin Q.-W., Oppenheim R.W., Bothwell M. (1994) Positive and negative effects of neurotrophins on the isthmo-optic nucleus in chick embryos. Neuron 12, 639–654 10.1016/0896-6273(94)90219-4 8155324

[29] Taylor A.R., Gifondorwa D.J., Robinson M.B., Strupe J.L., Prevette D., Johnson J.E. et al. (2012) Motoneuron programmed cell death in response to proBDNF. Dev. Neurobiol. 72, 699–712 10.1002/dneu.20964 21834083 PMC3233653

[30] Frade J.M., Barde Y.A. (1999) Genetic evidence for cell death mediated by nerve growth factor and the neurotrophin receptor p75 in the developing mouse retina and spinal cord. Development 126, 683–690 9895316 10.1242/dev.126.4.683

[31] Sedel F., Béchade C., Triller A. (1999) Nerve growth factor (NGF) induces motoneuron apoptosis in rat embryonic spinal cord in vitro. Eur. J. Neurosci. 11, 3904–3912 10.1046/j.1460-9568.1999.00814.x 10583479

[32] Pehar M., Cassina P., Vargas M.R., Castellanos R., Viera L., Beckman J.S. et al. (2004) Astrocytic production of nerve growth factor in motor neuron apoptosis: implications for amyotrophic lateral sclerosis. J. Neurochem. 89, 464–473 10.1111/j.1471-4159.2004.02357.x 15056289

[33] Harrington A.W., Leiner B., Blechschmitt C., Arevalo J.C., Lee R., Mörl K. et al. (2004) Secreted proNGF is a pathophysiological death-inducing ligand after adult CNS injury. Proc. Natl. Acad. Sci. U.S.A. 101, 6226–6230 10.1073/pnas.0305755101 15026568 PMC395951

[34] Sørensen B., Tandrup T., Koltzenburg M., Jakobsen J. (2003) No further loss of dorsal root ganglion cells after axotomy in p75 neurotrophin receptor knockout mice. J. Comp. Neurol. 459, 242–250 10.1002/cne.10625 12655507

[35] Tauris J., Gustafsen C., Christensen E.I., Jansen P., Nykjaer A., Nyengaard J.R. et al. (2011) Proneurotrophin-3 may induce Sortilin dependent death in inner ear neurons. Eur. J. Neurosci. 33, 622–631 10.1111/j.1460-9568.2010.07556.x 21261755 PMC3078644

[36] Carson C., Saleh M., Fung F.W., Nicholson D.W., Roskams A.J. (2005) Axonal dynactin p150Glued transports caspase-8 to drive retrograde olfactory receptor neuron apoptosis. J. Neurosci. 25, 6092–6104 10.1523/JNEUROSCI.0707-05.2005 15987939 PMC6725069

[37] Ibáñez C.F., Simi A. (2012) p75 neurotrophin receptor signaling in nervous system injury and degeneration: paradox and opportunity. Trends Neurosci. 35, 431–440 10.1016/j.tins.2012.03.007 22503537

[38] Yano H., Torkin R., Martin L.A., Chao M.V., Teng K.K. (2009) Proneurotrophin-3 Is a neuronal apoptotic ligand: evidence for retrograde-directed cell killing. J. Neurosci. 29, 14790–14802 10.1523/JNEUROSCI.2059-09.2009 19940174 PMC2824605

[39] Howe C.L., Mobley W.C. (2004) Signaling endosome hypothesis: a cellular mechanism for long distance communication. J. Neurobiol. 58, 207–216 10.1002/neu.10323 14704953

[40] Howe C.L., Mobley W.C. (2005) Long-distance retrograde neurotrophic signaling. Curr. Opin. Neurobiol. 15, 40–48 10.1016/j.conb.2005.01.010 15721743

[41] Cosker K.E., Courchesne S.L., Segal R.A. (2008) Action in the axon: generation and transport of signaling endosomes. Curr. Opin. Neurobiol. 18, 270–275 10.1016/j.conb.2008.08.005 18778772 PMC2693191

[42] Wu C., Cui B., He L., Chen L., Mobley W.C. (2009) The coming of age of axonal neurotrophin signaling endosomes. J. Proteomics 72, 46–55 10.1016/j.jprot.2008.10.007 19028611 PMC2677075

[43] Harrington A.W., Ginty D.D. (2013) Long-distance retrograde neurotrophic factor signalling in neurons. Nat. Rev. Neurosci. 14, 177–187 10.1038/nrn3253 23422909

[44] Yamashita N., Kuruvilla R. (2016) Neurotrophin signaling endosomes: biogenesis, regulation, and functions. Curr. Opin. Neurobiol. 39, 139–145 10.1016/j.conb.2016.06.004 27327126 PMC4987223

[45] Howe C.L., Valletta J.S., Rusnak A.S., Mobley W.C. (2001) NGF signaling from clathrin-coated vesicles. Neuron 32, 801–814 10.1016/S0896-6273(01)00526-8 11738027

[46] Chowdary P.D., Che D.L., Cui B. (2012) Neurotrophin signaling via long-distance axonal transport. Annu. Rev. Phys. Chem. 63, 571–594 10.1146/annurev-physchem-032511-143704 22404590

[47] Schmieg N., Menendez G., Schiavo G., Terenzio M. (2014) Signalling endosomes in axonal transport: travel updates on the molecular highway. Semin. Cell Dev. Biol. 27, 32–43 10.1016/j.semcdb.2013.10.004 24171925

[48] MacInnis B.L., Campenot R.B. (2002) Retrograde support of neuronal survival without retrograde transport of nerve growth factor. Science 295, 1536–1539 10.1126/science.1064913 11799202

[49] Wang T., Martin S., Nguyen T.H., Harper C.B., Gormal R.S., Martínez-Mármol R. et al. (2016) Flux of signalling endosomes undergoing axonal retrograde transport is encoded by presynaptic activity and TrkB. Nat. Commun. 7, 12976 10.1038/ncomms12976 27687129 PMC5427517

[50] Senger D.L., Campenot R.B. (1997) Rapid retrograde tyrosine phosphorylation of trkA and other proteins in rat sympathetic neurons in compartmented cultures. J. Cell Biol. 138, 411–421 10.1083/jcb.138.2.411 9230082 PMC2138199

[51] Bronfman F.C., Tcherpakov M., Jovin T.M., Fainzilber M. (2003) Ligand-induced internalization of the p75 neurotrophin receptor: a slow route to the signaling endosome. J. Neurosci. 23, 3209–3220 12716928 10.1523/JNEUROSCI.23-08-03209.2003 PMC6742322

[52] Saxena S., Howe C.L., Cosgaya J.M., Hu M., Weis J., Krüttgen A. (2004) Differences in the surface binding and endocytosis of neurotrophins by p75NTR. Mol. Cell. Neurosci. 26, 292–307 10.1016/j.mcn.2004.02.006 15207854

[53] Bronfman F.C., Escudero C.A., Weis J., Kruttgen A. (2007) Endosomal transport of neurotrophins: roles in signaling and neurodegenerative diseases. Dev. Neurobiol. 67, 1183–1203 10.1002/dneu.20513 17514710

[54] Hibbert A.P., Kramer B.M.R., Miller F.D., Kaplan D.R. (2006) The localization, trafficking and retrograde transport of BDNF bound to p75NTR in sympathetic neurons. Mol. Cell. Neurosci. 32, 387–402 10.1016/j.mcn.2006.06.001 16843677

[55] Escudero C.A., Lazo O.M., Galleguillos C., Parraguez J.I., Lopez-Verrilli M.A., Cabeza C. et al. (2014) The p75 neurotrophin receptor evades the endolysosomal route in neuronal cells, favouring multivesicular bodies specialised for exosomal release. J. Cell Sci. 127, 1966–1979 10.1242/jcs.141754 24569882 PMC4004974

[56] Deinhardt K., Reversi A., Berninghausen O., Hopkins C.R., Schiavo G. (2007) Neurotrophins redirect p75NTR from a clathrin-independent to a clathrin-dependent endocytic pathway coupled to axonal transport. Traffic 8, 1736–1749 10.1111/j.1600-0854.2007.00645.x 17897318

[57] Butowt R., von Bartheld C.S. (2009) Fates of neurotrophins after retrograde axonal transport: phosphorylation of p75NTR Is a sorting signal for delayed degradation. J. Neurosci. 29, 10715–10729 10.1523/JNEUROSCI.2512-09.2009 19710323 PMC2761711

[58] Deinhardt K., Salinas S., Verastegui C., Watson R., Worth D., Hanrahan S. et al. (2006) Rab5 and Rab7 control endocytic sorting along the axonal retrograde transport pathway. Neuron 52, 293–305 10.1016/j.neuron.2006.08.018 17046692

[59] Ziv N.E., Spira M.E. (1995) Axotomy induces a transient and localized elevation of the free intracellular calcium concentration to the millimolar range. J. Neurophysiol. 74, 2625–2637 8747220 10.1152/jn.1995.74.6.2625

[60] Mandolesi G., Madeddu F., Bozzi Y., Maffei L., Ratto G.M. (2004) Acute physiological response of mammalian central neurons to axotomy: ionic regulation and electrical activity. FASEB J. 18, 1934–1936 15451889 10.1096/fj.04-1805fje

[61] Bradke F., Fawcett J.W., Spira M.E. (2012) Assembly of a new growth cone after axotomy: the precursor to axon regeneration. Nat. Rev. Neurosci. 13, 183–193 22334213 10.1038/nrn3176

[62] Yan D., Jin Y. (2012) Regulation of DLK-1 kinase activity by calcium-mediated dissociation from an inhibitory isoform. Neuron 76, 534–548 10.1016/j.neuron.2012.08.043 23141066 PMC3508676

[63] Ghosh A.S., Wang B., Pozniak C.D., Chen M., Watts R.J., Lewcock J.W. (2011) DLK induces developmental neuronal degeneration via selective regulation of proapoptotic JNK activity. J. Cell Biol. 194, 751–764 10.1083/jcb.201103153 21893599 PMC3171129

[64] Itoh A., Horiuchi M., Wakayama K., Xu J., Bannerman P., Pleasure D. et al. (2011) ZPK/DLK, a mitogen-activated protein kinase kinase kinase, is a critical mediator of programmed cell death of motoneurons. J. Neurosci. 31, 7223–7228 10.1523/JNEUROSCI.5947-10.2011 21593306 PMC3138193

[65] Cavalli V., Kujala P., Klumperman J., Goldstein L.S.B. (2005) Sunday driver links axonal transport to damage signaling. J. Cell Biol. 168, 775–787 10.1083/jcb.200410136 15738268 PMC2171809

[66] Shin J.E., Cho Y., Beirowski B., Milbrandt J., Cavalli V., DiAntonio A. (2012) Dual leucine zipper kinase is required for retrograde injury signaling and axonal regeneration. Neuron 74, 1015–1022 10.1016/j.neuron.2012.04.028 22726832 PMC3383631

[67] Mok S.-A., Lund K., Campenot R.B. (2009) A retrograde apoptotic signal originating in NGF-deprived distal axons of rat sympathetic neurons in compartmented cultures. Cell Res. 19, 546–560 10.1038/cr.2009.11 19188931

[68] Troy C.M., Friedman J.E., Friedman W.J. (2002) Mechanisms of p75-mediated death of hippocampal neurons: role of caspases. J. Biol. Chem. 277, 34295–34302 10.1074/jbc.M205167200 12097334

[69] Geden M.J., Deshmukh M. (2016) Axon degeneration: context defines distinct pathways. Curr. Opin. Neurobiol. 39, 108–115 10.1016/j.conb.2016.05.002 27197022 PMC4987202

[70] Unsain N., Higgins J.M., Parker K.N., Johnstone A.D., Barker P.A. (2013) XIAP regulates caspase activity in degenerating axons. Cell Rep. 4, 751–763 10.1016/j.celrep.2013.07.015 23954782

[71] Simon D.J., Weimer R.M., McLaughlin T., Kallop D., Stanger K., Yang J. et al. (2012) A caspase cascade regulating developmental axon degeneration. J. Neurosci. 32, 17540–17553 10.1523/JNEUROSCI.3012-12.2012 23223278 PMC3532512

[72] Cusack C.L., Swahari V., Henley W.H., Ramsey J.M., Deshmukh M. (2013) Distinct pathways mediate axon degeneration during apoptosis and axon-specific pruning. Nat. Commun. 4, 1876 10.1038/ncomms2910 23695670 PMC4183061

[73] Simon D.J., Pitts J., Hertz N.T., Yang J., Yamagishi Y., Olsen O. et al. (2016) Axon degeneration gated by retrograde activation of somatic pro-apoptotic signaling. Cell 164, 1031–1045 10.1016/j.cell.2016.01.032 26898330 PMC4785881

[74] Singh K.K., Park K.J., Hong E.J., Kramer B.M., Greenberg M.E., Kaplan D.R. et al. (2008) Developmental axon pruning mediated by BDNF-p75NTR-dependent axon degeneration. Nat. Neurosci. 11, 649–658 10.1038/nn.2114 18382462

[75] Park K.J., Grosso C.A., Aubert I., Kaplan D.R., Miller F.D. (2010) p75NTR-dependent, myelin-mediated axonal degeneration regulates neural connectivity in the adult brain. Nat. Neurosci. 13, 559–366 10.1038/nn.2513 20348920

[76] Perlson E., Jeong G-B, Ross J.L., Dixit R., Wallace K.E., Kalb R.G. et al. (2009) A switch in retrograde signaling from survival to stress in rapid-onset neurodegeneration. J. Neurosci. 29, 9903–9917 10.1523/JNEUROSCI.0813-09.2009 19657041 PMC3095444

[77] Lowry K.S., Murray S.S., McLean C.A., Talman P., Mathers S., Lopes E.C. et al. (2001) A potential role for the p75 low-affinity neurotrophin receptor in spinal motor neuron degeneration in murine and human amyotrophic lateral sclerosis. Amyotroph. Lateral Scler. Other Motor Neuron Disord. 2, 127–134 10.1080/146608201753275463 11771768

[78] Seeburger J.L., Tarras S., Natter H., Springer J.E. (1993) Spinal cord motoneurons express p75NGFR and p145trkB mRNA in amyotrophic lateral sclerosis. Brain Res. 621, 111–115 10.1016/0006-8993(93)90304-6 8221061

[79] Küst B.M., Brouwer N., Mantingh I.J., Boddeke H., Copray J. (2003) Reduced p75NTR expression delays disease onset only in female mice of a transgenic model of familial amyotrophic lateral sclerosis. Amyotroph. Lateral Scler. Other Motor Neuron Disord. 4, 100–105 14506941

[80] Syroid D.E., Maycox P.J., Soilu-Hänninen M., Petratos S., Bucci T., Burrola P. et al. (2000) induction of postnatal Schwann cell death by the low-affinity neurotrophin receptor in vitro and after axotomy. J. Neurosci. 20, 5741–5747 10908614 10.1523/JNEUROSCI.20-15-05741.2000 PMC6772552

[81] Hanbury R., Charles V., Chen E-Y, Leventhal L., Rosenstein J.M., Mufson E.J. et al. (2002) Excitotoxic and metabolic damage to the rodent striatum: role of the p75 neurotrophin receptor and glial progenitors. J. Comp. Neurol. 444, 291–305 10.1002/cne.10104 11891644

[82] Schachtrup C., Ryu J.K., Mammadzada K., Khan A.S., Carlton P.M., Perez A. et al. (2015) Nuclear pore complex remodeling by p75NTR cleavage controls TGF-β signaling and astrocyte functions. Nat. Neurosci. 18, 1077–1080 10.1038/nn.4054 26120963 PMC4878404

[83] Turner B.J., Murray S.S., Piccenna L.G., Lopes E.C., Kilpatrick T.J., Cheema S.S. (2004) Effect of p75 neurotrophin receptor antagonist on disease progression in transgenic amyotrophic lateral sclerosis mice. J. Neurosci. Res. 78, 193–199 10.1002/jnr.20256 15378612

[84] Pehar M., Vargas M.R., Cassina P., Barbeito A.G., Beckman J.S., Barbeito L. (2006) Complexity of astrocyte–motor neuron interactions in amyotrophic lateral sclerosis. Neurodegener. Dis. 2, 139–146 10.1159/000089619 16909019

[85] Matusica D., Alfonsi F., Turner B.J., Butler T.J., Shepheard S.R., Rogers M-L et al. (2016) Inhibition of motor neuron death in vitro and in vivo by a p75 neurotrophin receptor intracellular domain fragment. J. Cell Sci. 129, 517–530 10.1242/jcs.173864 26503157

[86] Turner B.J., Cheah I.K., Macfarlane K.J., Lopes E.C., Petratos S., Langford S.J. et al. (2003) Antisense peptide nucleic acid-mediated knockdown of the p75 neurotrophin receptor delays motor neuron disease in mutant SOD1 transgenic mice. J. Neurochem. 87, 752–763 10.1046/j.1471-4159.2003.02053.x 14535957

[87] Perlson E., Maday S., Fu M., Moughamian A.J., Holzbaur E.L.F. (2010) Retrograde axonal transport: pathways to cell death. Trends Neurosci. 33, 335–344 10.1016/j.tins.2010.03.006 20434225 PMC2902719

[88] Encalada S.E., Goldstein L.S.B. (2014) Biophysical challenges to axonal transport: motor-cargo deficiencies and neurodegeneration. Annu. Rev. Biophys. 43, 141–169 10.1146/annurev-biophys-051013-022746 24702007

